# Mild heat treatment in vitro potentiates human adipose stem cells: delayed aging and improved quality for long term culture

**DOI:** 10.1186/s40824-023-00448-w

**Published:** 2023-11-27

**Authors:** Chiseon Ryu, Minseo Lee, Jae Young Lee

**Affiliations:** School of Materials Science and Engineering, Gwangju Institute of Science and Technology, Gwangju, 61005 Republic of Korea

**Keywords:** Heat treatment, Mesenchymal stem cell, Stemness, Aging, In vitro culture

## Abstract

**Background:**

Mesenchymal stem cells (MSCs) have gained significant attention for diverse biomedical applications, including cell-based therapy. Hence, in vitro expansion of MSCs is critical; however, in vitro MSC culture, especially long-term culture, inevitably leads to significant loss of stemness, growth, and differentiation potential.

**Method:**

Effects of mild heat treatment (HT) conditions (temperature, duration, and repetition) on the characteristics of adipose tissue-derived MSCs in vitro were systematically investigated. Characteristics of the MSCs subjected to the predetermined HT conditions (41 or 44ºC, 1 h, and 2X HT) were first analyzed in a single passage using various assays. In addition, the feasibility of HT for long-term MSC culture was studied. The RNA sequencing analyses were performed to elucidate the mechanism of HT effects on MSCs.

**Results:**

A comprehensive exploration of various HT conditions revealed that specific mild HT at 41ºC or 44ºC for 1 h upregulated the expression of heat shock proteins and stemness markers and enhanced differentiation potentials. Furthermore, periodic mild HT extended the maintenance of growth rate and stemness of MSCs up to an additional 10 passages, which substantially retarded their spontaneous aging during subsequent in vitro culture. RNA sequencing analyses unveiled that HT downregulated genes associated with aging and apoptosis.

**Conclusion:**

Our study successfully demonstrated that mild HT of MSCs has positive effects on their application in various biomedical fields, enhancing their capabilities and slowing down the aging process.

**Graphical Abstract:**

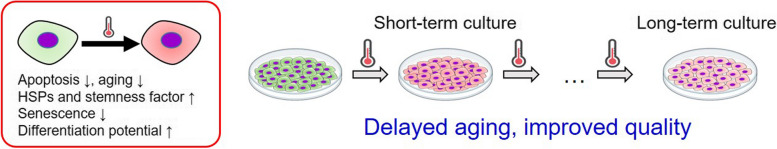

**Supplementary Information:**

The online version contains supplementary material available at 10.1186/s40824-023-00448-w.

## Introduction

Mesenchymal stem cells (MSCs) have been extensively studied recently for various biomedical applications, including cell-based therapies, owing to their self-renewal, differentiation, and therapeutic potential. In addition, the lack of ethical concerns and convenient isolation of MSCs from various tissues have made them an attractive source [1]. MSCs can differentiate into multiple lineages (*e.g.*, bone, cartilage, and adipose tissues). These cells secrete various bioactive molecules which exert immunomodulatory, anti-inflammatory, and angiogenic effects [2]. MSCs are an effective cell source for tissue engineering. In addition, exosomes and cellular vesicles from MSCs have also been studied as promising therapeutic agents for tissue engineering [3]. Accordingly, the in vitro expansion of MSCs possessing the abovementioned characteristics is a prerequisite; however, in vitro MSC cultures inevitably lead to a considerable loss of their original characteristics with incubation especially for long-term culture. MSCs during in vitro culture eventually lose their stemness, growth rate, and differentiation potentials, which is commonly called as ‘aging’ [4]. For instance, the growth rate of bone marrow-derived MSCs dramatically decreases, and they become morphologically enlarged and flattened with successive in vitro passages [4, 5]. Most MSC manufacturers and laboratories cautiously indicate a limit for the number of MSC passages for the optimum quality control of MSC characteristics. However, maintaining optimum MSC quality during in vitro cell culture remains challenging [6].

Hence, there have been extensive efforts to reduce spontaneous aging processes and produce a large number of high-quality MSCs by developing novel in vitro culture conditions such as hypoxia, hyperthermia, supplementation of media components, and the use of different culture substrates [7]. Especially, nonbiochemical cues (*e.g.*, hypoxia and hyperthermia) can be easily and effectively applied to alter cellular behavior during in vitro cell culture. For example, hypoxia, which mimics oxygen-limiting in vivo conditions (< 3%), was found to promote the growth rate and maintain the stemness of MSCs during in vitro culture [8]. In addition, hyperthermia, which is a mild heat treatment (HT), can influence cellular responses without causing cell death [9]. HT has been widely employed for pain relief and blood circulation [10]. These marginal temperature elevations can affect cellular behavior (*e.g.*, growth and differentiation) and various intracellular signaling pathways, including the upregulation of heat shock proteins (HSPs) [11]. Multiple studies have examined the effects of mild HT on MSC behavior; however, the results have been controversial. For instance, the in vitro culture of bovine MSCs at 42ºC for 1 h induced apoptosis and premature senescence [12]. In contrast, HT reduced apoptosis and enhanced the growth rate of rat bone marrow derived-MSCs [13]. These contrasting findings might be attributed to the different experimental HT conditions (*e.g.*, temperature, duration, and frequency) and cell types. More importantly, the majority of research has explored the therapeutic effects of single heat treatment on MSCs and focused on cellular behaviors in a short time period, leaving a lack of investigation into long-term MSC culture and the underlying mechanisms [9, 13, 14]. Accordingly, a systematic investigation is required to clearly elucidate MSC responses to HT and identify specific HT conditions for a long-term in vitro MSC culture [15].

This study aimed to develop HT-based culture conditions for the large production of high-quality adipose-derived MSCs (AD-MSCs) and elucidate the intracellular mechanism. AD-MSCs offer several benefits as a cell source for biomedical engineering applications, including simple isolation from adipose tissue, high differentiation potential, and the secretion of various growth factors and cytokines [16]. We evaluated various HT conditions (such as temperature, duration, and repetition) with respect to cell viability, stemness maintenance, and differentiation potential. We initially examined the expression of HSPs as a primary indicator in response to HT, owing to their essential roles in cellular behavior, such as survival and growth [11]. For example, *HSP27* overexpression in MSCs enhanced cell survival, reduced apoptosis, and improved therapeutic efficacy [17]. Importantly, we applied successive HT to MSCs for up to 10 passages to examine their long-term effects and potential to produce large quantities of high-quality MSCs. Moreover, the mechanisms by which HT affected MSCs during culture were examined using total RNA sequencing.

## Materials and methods

### Materials

Minimum Essential Medium-alpha (MEM-α), Dulbecco’s phosphate buffered saline (DPBS), fetal bovine serum (FBS) antibiotic–antimycotic, trypsin, StemPro Adipogenesis Differentiation Medium, StemPro Osteogenesis Differentiation Medium, StemPro Chondrogenesis Differentiation Medium were purchased from Gibco (Grand Island, NY, USA). Human adipose-derived MSCs were obtained from PromoCell (Heidelberg, Germany). Chloroform, ethanol, 1% crystal violet solution, Triton-X, Oil Red O, isopropanol, Alizarin Red S, Alcian blue, cetylpyridinium chloride (CPC), acetic acid solution, trypan blue, and formaldehyde solution were purchased from Sigma-Aldrich (St. Louis, MO, USA). High-Capacity cDNA Reverse Transcription Kit and PowerUp SYBR Green Master Mix were purchased from Applied Biosystems (Foster City, CA, USA). TRIzol reagent, LIVE/DEAD viability kit, CD14-PE, CD34-PE, CD45-PE, CD73-PE, CD90-PE, CD105-PE, and mouse IgG1 kappa Isotype Control-PE were purchased from Invitrogen (Carlsbad, CA, USA). AccuPrep® Genomic DNA Extraction Kit was purchased from Bioneer (Daejeon, Korea), WST solution (EZ-cytox) was purchased from DoGenBio (Seoul, Korea), LDH cytotoxicity detection kit was purchased from Takara (Kusatsu, Japan), Senescence β-galactosidase Staining kit was purchased from Cell Signaling Technology (Danvers, MA, USA).

### Mesenchymal stem cell culture

Human AD-MSCs were cultured in minimum essential medium-α supplemented with 10% fetal bovine serum and 1% antibiotic–antimycotic, and incubated in humidified air with 5% CO_2_ at 37ºC. The medium was replaced every 2 d, and the cells were subcultured at 80% confluency. AD-MSCs at passage five were used in all experiments unless otherwise noted. Bright-field images were acquired using an optical microscope (DMI3000B; Leica).

### Mild heat treatment

AD-MSCs at passage five were seeded in a 12-well plate at a cell density of 2.5 × 10^4^/well and incubated for 24 h in a 37ºC humidified incubator. Cells were divided into control and HT groups. The control group was incubated in a 37ºC incubator during the whole culture period. For the HT group, AD-MSCs were incubated in a 37ºC incubator for 48 h, moved in an incubator set at specific temperature (39, 41, 44, or 47ºC) for specific time (0.5, 1, or 2 h), and then moved back to the 37ºC incubator. For 2X HT, this procedure was repeated on the following day in the same manner. At least three experiments were performed on different days (*n* = 3—5).

### Water-soluble tetrazolium salt assay

The metabolic activity of the cells was quantified using a water-soluble tetrazolium salt (WST)-1 assay. WST solution was mixed with the growth medium at a volume ratio of 1:10. Culture media in each well was replaced with the mixed solution (500 μL) and incubated for additional 1 h. Then, the absorbance of the cultured solution was measured at 450 nm using a microplate reader (Varioskan LUX, Thermo Fisher). Relative metabolic activity (%) was calculated using the following formula:

$$Relative\;metabolic\;activity\;\left(\%\right)=\frac{Absorbance(sample)}{Absorbance(control)} \times 100$$

### LDH assay

LDH release from each sample was quantified using an LDH cytotoxicity detection kit, following the manufacturer’s protocol. The culture media from each group were collected on day 4 and mixed with the catalyst and dye solutions. The absorbance was measured at 490 nm using a microplate reader. The LDH release (%) was calculated using the following formula:

$$LDH\;release \left(\%\right)=\frac{Absorbance(sample)-Absorbance(low\;control)}{Absorbance(high\;control)-Absorbance(low\;control)} \times 100$$

where high and low controls indicate maximal LDH release (treated with Triton-X) and minimal LDH release (treated with culture media without cells), respectively.

### Live/dead assay

Cells were detached by treatment with 0.05% trypsin and then treated with calcein AM and EthD-1 solutions using a LIVE/DEAD viability kit, according to the manufacturer’s protocol. After staining, the cells were washed twice with Dulbecco's phosphate-buffered saline (DPBS) and fluorescence was determined using a flow cytometer (FACSCanto II, BD Biosciences). The percentage of live cells was obtained from the area portion in flow cytometry data analyzed for 10,000 gated-cell events using the FlowJo software (BD Biosciences, Franklin Lakes, NJ, version 10.8.1).

### RNA isolation and quantitative RT-PCR

Total RNA was isolated from individual experimental groups using TRIzol reagent. Complementary DNA (cDNA) was synthesized from isolated mRNA using a High-Capacity cDNA Reverse Transcription Kit, according to the manufacturer’s protocol. Quantitative PCR (qRT-PCR) was performed using Power SYBR Green PCR Master Mix with a StepOnePlus Real-Time PCR System (Applied Biosystems), according to the manufacturer’s instructions. Target gene expression was normalized to that of glyceraldehyde-3-phosphate dehydrogenase for quantification. The primer sequences used for the qRT-PCR analysis are listed in Table S1.

### Colony formation assay

Cells in each group were detached by 0.05% trypsin treatment, seeded in 6-well plates at a seeding density of 100 cells/well, and incubated in a 37ºC humidified incubator for 3 weeks to form colonies. Colonies were fixed with 4% formaldehyde and stained with 0.5% crystal violet. The number of purple dots in each well was counted and expressed as the number of colonies per well.

### Cell senescence

Senescence of the cultured AD-MSCs was analyzed using the Senescence β-galactosidase Staining kit. MSCs were seeded in 12-well plates and cultured under different HT conditions, as described above. Cells were stained according to the manufacturer’s protocol. The images were acquired using an optical microscope and the portion of β-gal stained-blue area in each image was analyzed using the ImageJ software (NIH, Bethesda, MD, USA, version 1.25p).

### Fluorescence-activated cell sorting analysis

The expression of various AD-MSC surface antigens were evaluated using flow cytometry. After culturing, the cells were detached using 0.05% trypsin. The cells were then washed with culture media and DPBS, and stained with monoclonal antibodies against CD14-PE, CD34-PE, CD45-PE, CD73-PE, CD90-PE, and CD105-PE for 30 min at 25ºC. Corresponding isotype control cells were stained with mouse IgG1 kappa isotype control phycoerythrin. The cells were washed thrice with DPBS and analyzed using flow cytometry. Population (%) and MFI were obtained from the flow cytometry data using FlowJo software for 10,000 gated cell events. MFI was normalized to that of the control group to determine the relative MFI.

### In vitro differentiation

AD-MSCs from each group at a specific passage were obtained by trypsin treatment, seeded in 12-well plates at specific cell densities (described below) and incubated in growth medium in a humidified incubator for 24 h. The plates were divided into three groups for individual differentiation, and the medium was replaced with differentiation medium. The medium was replaced every three days.

For adipogenic differentiation, MSCs were seeded in 12-well plates at a density of 4 × 10^4^ cells/well and incubated in StemPro adipogenic differentiation medium for 7 days. Each well was stained with Oil Red O according to the manufacturer's protocol. Stained images were acquired using an optical microscope. To quantify the lipids produced, the stain was dissolved in 100% isopropanol. Absorbance of the extract was measured at 512 nm using a microplate reader. For osteogenic differentiation, MSCs were seeded in 12-well plates at a density of 2 × 10^4^ cells/well and incubated in StemPro osteogenic differentiation medium for 3 weeks. Each well was stained with Alizarin Red S according to the manufacturer's protocol. Stained images were acquired using an optical microscope. The stain was dissolved in 10% cetylpyridinium chloride solution, and the absorbance of the extract solution was measured at 556 nm using a microplate reader. For chondrogenic differentiation, MSCs were seeded in 12-well plates at a density of 8 × 10^4^ cells/well and incubated in the StemPro chondrogenesis differentiation medium for 2 weeks. Each well was stained with Alcian blue, and the stain was dissolved in a 3% acetic acid solution. Absorbance of the extract was measured at 600 nm using a microplate reader. After in vitro differentiation, lineage-specific gene expression was analyzed using qRT-PCR.

### Long-term MSC culture

Long-term cultures were performed to study the effects of HT on MSC characteristics, including the lasting effects of HT alone and the effects of periodic HT. The passage five MSCs were seeded into the control and HT groups at a cell density of 2.5 × 10^4^ per well and subcultured at 80% confluency. After passaging, the MSCs were noted as P + 1. For multiple passages (*n*), the MSCs were denoted as P + *n*. After each subculture, the trypsinized cells were stained with trypan blue and the cell number was counted using a hemocytometer. The cells of each group were diluted to a concentration (0.5 × 10^4^ cells/mL) and seeded into 12-well plates at a cell density of 0.5 × 10^4^ per well for additional subculture. For the lasting effect experiments, cells were successively passaged in the same manner without HT into five additional subcultures (P + 6). For the periodic HT experiments, the cells were cultured with HT (37ºC (control), 44ºC, or 44ºC; 1 h per day; 2X HT) at every passage. Subsequent passages with HT were denoted as P + *n*(HT), where *n* indicates the number of passages. The cumulative cell number was determined as the sum of the average cell numbers recorded at each passage. Doubling time was calculated using the exponential curve equation with the initial cell density (seeding density), final cell density (average cell number), and total incubation time at each passage.

### Telomere length measurement

The telomere length was measured for MSCs in each group at P + 10(HT). The genomic DNA was extracted from MSCs using AccuPrep® Genomic DNA Extraction Kit according to the manufacturer’s protocol. Relative telomere length was determined using qRT-PCR, according to previously reported methods [18, 19]. qRT-PCR determines the cycle threshold (Ct) value of telomeric DNA and the single-copy control gene, and the relative telomere length was calculated based on the ΔΔCt method. The primer sequences are listed in Table S1.

### Total RNA sequencing

For total RNA sequencing, 1 × 10^6^ cells per sample from the control (37ºC) and HT 44ºC groups were collected. Quant-Seq analysis of each sample was performed in duplicates (Ebiogen, Seoul, Korea). The results were analyzed using the ExDEGA software (Ebiogen, Seoul, Korea). Differentially expressed genes with fold changes of > 2 and* p* values < 0.05 were selected. Based on the 101 genes identified above, related gene ontologies were identified using DAVID (http://david.abcc.ncifcrf.gov/) and QuickGO (https://www.ebi.ac.uk/QuickGO/). Ten major gene ontologies were selected and are listed. The 54 genes involved in the gene ontologies were subsequently plotted as a heat map using MultiExperiment Viewer software (MeV; J. Craig Venter Institute, Rockville, MD, USA, version 4.9.0).

### Statistical analyses

The experiments were performed in triplicates and the experimental value was indicated as the mean ± standard deviation unless otherwise stated. Differences among samples were compared using one-way analysis of variance with Tukey’s post hoc comparison at a significance level of *p* < 0.05.

## Results

### Hyperthermia conditions

**Fig. 1:**
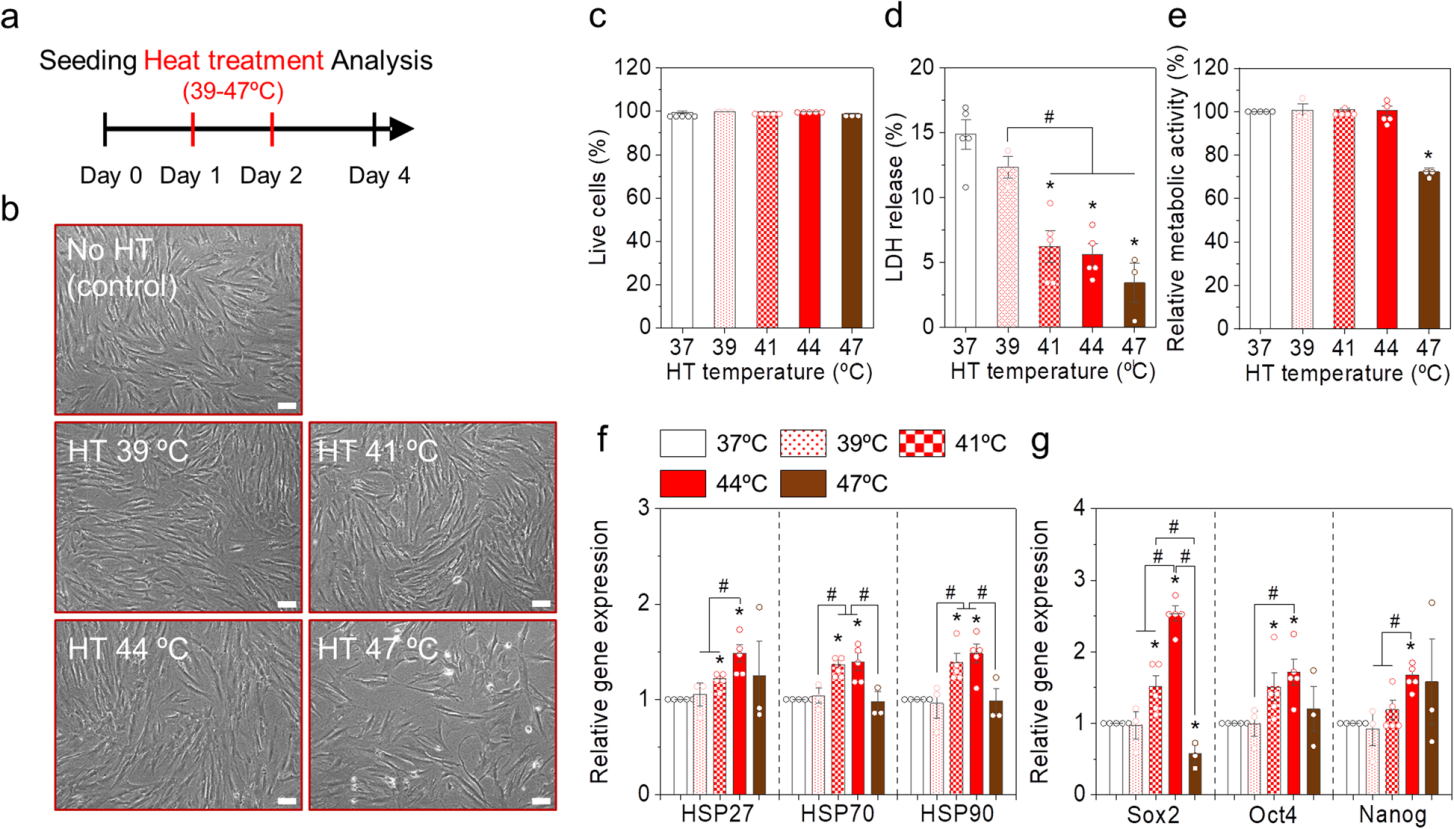
Mild heat treatment (HT) of human adipose tissue-derived mesenchymal stem cells (AD-MSCs) in a single passage at different temperatures. **a** Schematic representation of the experimental design. **b** Optical micrographs of the MSCs in each group on day 4. Scale bar is 200 μm. **c** Live cell percentage, **d** lactate dehydrogenase (LDH) levels, and **e** relative metabolic activities of the MSCs in each group. Gene expression levels of **f** heat shock proteins (HSPs) and **g** stemness markers. Gene expression levels were normalized with the control (37ºC). Error bars represent the average ± the standard error of the mean from three to five experiments. *p < 0.05 compared to the control group, and ^#^*p* < 0.05 compared to two groups

We systematically examined various parameters to establish optimal HT conditions for in vitro MSC culture in a single passage (Fig. 1). We hereby focused on identifying HT conditions that significantly induced beneficial heat shock responses without hindering cell growth by evaluating gene expressions (HSPs and stemness transcription factors) and cell viability. First, MSCs were thermally stimulated at different temperatures ranging from 37–47ºC for 1 h on Day 1 and Day 2 (twice HT) (Fig. 1a). MSCs in all groups exhibited spindle-shaped, fibroblast-like morphologies (Fig. 1b). The live cell proportions in all HT groups were not significantly different from those in the control group (Fig. 1c), and lactate dehydrogenase (LDH) levels, which are indicative of apoptotic cells, were significantly lower in the HT groups than those in the control group (Fig. 1d). Notably, LDH levels in the HT groups at higher temperatures (41, 44, and 47ºC) (3.4–6.2%) were significantly lower than those in the control (14.9 ± 1.1%) and 39ºC HT (12.4 ± 0.9%) groups. The metabolic activities of the MSCs stimulated at 39, 41, and 44ºC were similar to those of the control, whereas the metabolic activity of MSCs in the 47ºC HT group was 0.73-fold lower than that in the control group (Fig. 1e). Results indicated that mild HT (39–44ºC) did not impair cell viability or the metabolic activity of MSCs. However, HT 47 ºC group exhibited lower metabolic activity and relatively lower confluency in optical image than other groups, which appeared to be attributed to lower cellular energy state and growth rate while not triggering apoptosis or cell death [20].

Subsequently, we analyzed the expression of HSP genes (*HSP27, HSP70*, and *HSP90*) and stemness-associated genes (*SOX2*, *OCT4*, and *NANOG*). Overall, HT at 41–47ºC significantly upregulated the expression of HSPs (Fig. 1f). The MSCs stimulated at 41 and 44ºC consistently exhibited higher mRNA expression of the tested HSPs than those 37, 39, and 47ºC, indicating that HT at 41 and 44ºC are optimal to upregulate HSP expression. Similarly, the HT groups at 41 and 44ºC showed significantly higher expression of *SOX2*, *OCT4*, and *NANOG*, which are master transcription factors essential for maintaining stemness in MSCs [21], compared to other groups (Fig. 1g). The expression of these stemness genes was slightly higher for the 44ºC HT group than that for the 41ºC HT group. Overall, our results identified that HT at 41 and 44ºC for 1 h can consistently increase the expression of HSPs and the stemness-associated transcription factors without altering cell viability. These two temperatures were used in the subsequent experiments.

**Fig. 2:**
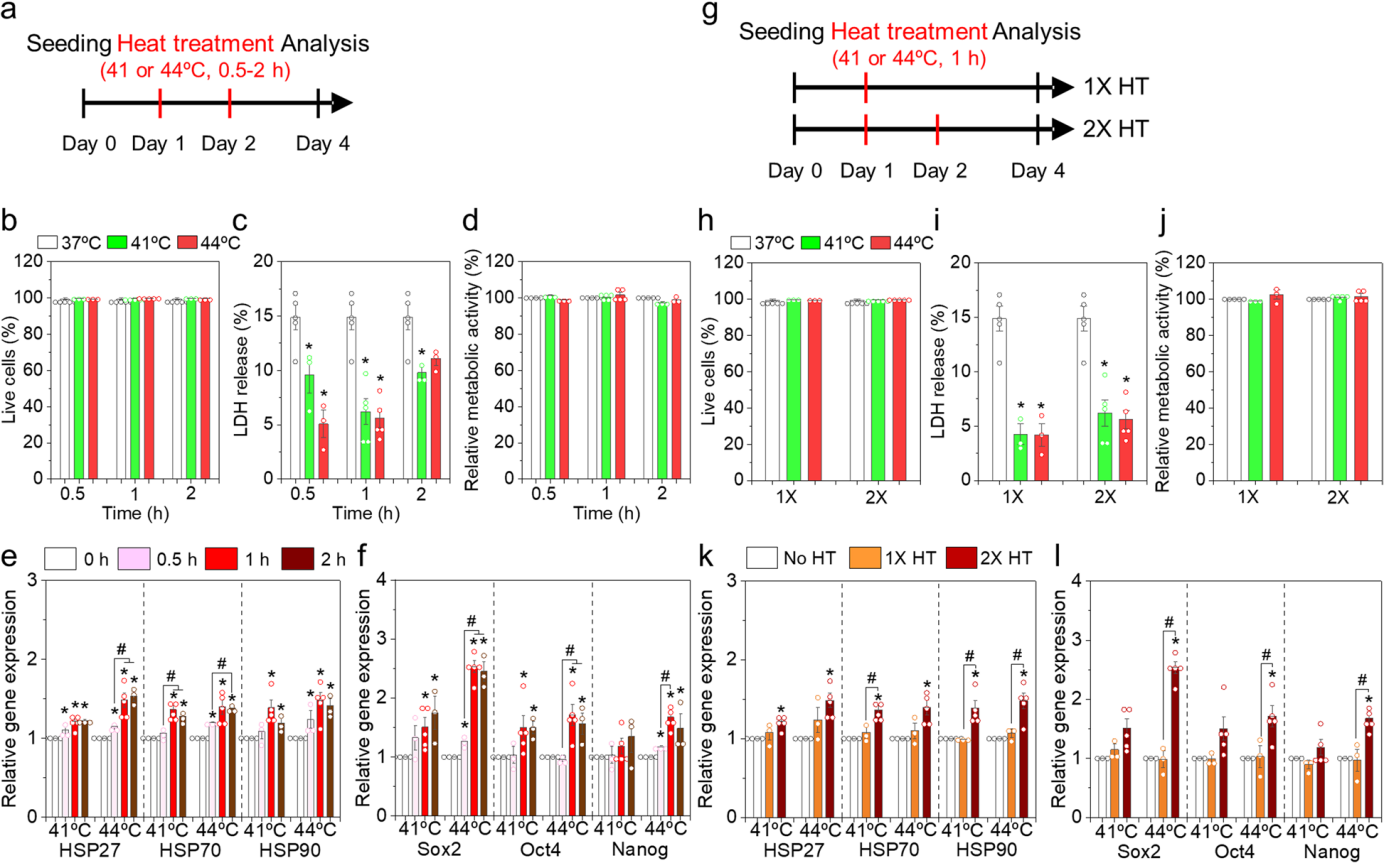
Mild HT of AD-MSCs for varying durations (**a** – **f**) and repetition (**g** – **l**) in a single passage. **a** An experimental scheme to study the effects of HT duration. **b** Live cell percentage, **c** LDH levels, and **d** relative metabolic activities of MSCs in each group. Gene expression levels of **e** HSPs and **f** stemness markers. **g** An experimental scheme to study the effects of HT repetition. **h** Live cell percentage, **i** LDH levels, and **j** relative metabolic activities of MSCs in each group. Gene expression levels of **k** HSPs and **l** stemness markers. Gene expression levels were normalized with the control (37ºC). Error bars represent the average ± the standard error of the mean from three to five experiments. *p < 0.05 compared to the control group, and ^#^*p* < 0.05 compared to two groups

Further, we explored the effects of HT duration (0.5–2 h) at 41 or 44ºC on MSC viability and gene expression in a single passage (Fig. 2a). No morphological differences were observed between the control and HT groups, and all MSCs exhibited a typical spindle shape (Fig. S1). The proportions of live cells in all the HT groups were almost 100%, which were similar to those in the control group (Fig. 2b). MSCs stimulated at 41ºC for 0.5–2 h and at 44ºC for 0.5 h or 1 h secreted significantly lower LDH to the medium than MSCs in the control group (Fig. 2c). Metabolic activities were similar in all the tested groups (Fig. 2d). Our results suggest that the tested HT conditions (41 or 44ºC for 0.5–2 h) did not cause significant cytotoxicity. The gene expression levels of HSPs were found to substantially increase in response to HT treatment. HSP gene expression gradually increased with increase in HT duration (Fig. 2e). HT temperature (41 or 44ºC) did not significantly influence gene expression of *HSP70* and *HSP90*, but in case of 1 or 2 h HT duration, *HSP27* gene expression was higher in the 44ºC HT groups than that in 41ºC groups. In addition, the expression of stemness genes was higher in the HT groups, especially stimulated for 1 – 2 h, than that in the control group (Fig. 2f). Overall, gene expressions of *SOX2* and *NANOG* were slightly higher in the 44ºC HT groups than those in the 41ºC HT groups. HT for 2 h at either 41 or 44ºC did not substantially increase the expression of these stemness genes compared to those at HT for 1 h. Altogether, 1 h of HT at 41 and 44ºC was determined to be optimal to thermally simulate MSCs and alter the expression of HSPs and stem cell transcription factors.

We further studied the effects of HT repetition under predetermined conditions (1 h at 41 or 44ºC). A single HT (1X HT) was conducted on day one, whereas a double HT (2X HT) was conducted on days one and two (Fig. 2g). Duplication with HT did not affect cell viability or metabolic activity (Fig. 2h, i, and j). MSCs with 2X HT at 41 or 44ºC released slightly higher levels of LDH than those with 1X HT; however, their levels were still lower than those in the control with a decrease of 0.42- and 0.38-fold, respectively. Interestingly, 1X HT at both 41 or 44ºC did not significantly promote the gene expression of HSPs and stemness-associated transcription factors. In contrast, 2X HT significantly upregulated HSP and stemness gene expression, suggesting the importance of repeated HTs in modulating intracellular responses and maintaining stemness (Fig. 2k and l).

### Effects of HT on the stemness of AD-MSCs

**Fig. 3:**
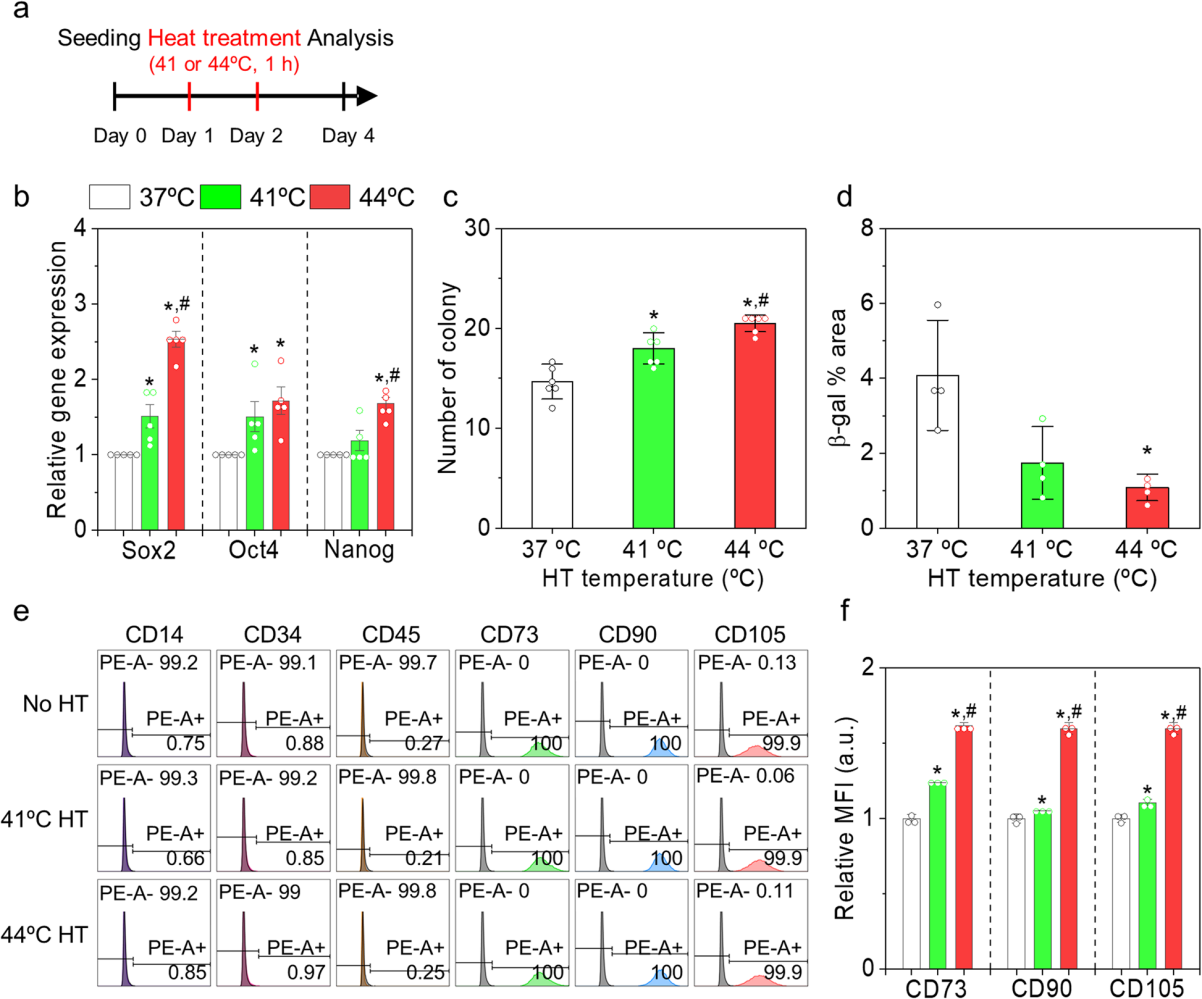
Effects of mild HT on stemness of human AD-MSCs. **a** Schematic representation of the experimental design. **b** Relative gene expression of *SOX2*, *OCT4*, and *NANOG*, **c** the number of colonies, and **d** β-galactosidase (β-gal) stained area in each group. **e** Flow cytometry of the MSCs stained with negative markers (CD14, CD34, and CD45) and positive markers (CD73, CD90, and CD105). **f** Mean fluorescence intensity (MFI) of the positive marker-stained MSCs in each group. *p < 0.05 compared to the control group, and ^#^*p* < 0.05 compared to the HT 41ºC group

We further explored characteristics of the MSCs subjected to the predetermined HT conditions (41 or 44ºC, 1 h, and 2X HT) in a single passage (Fig. 3a) and evaluated the quality of MSCs using various assays. As previously mentioned, the gene expression of stemness transcription factors was substantially upregulated in the HT groups (Fig. 3b). Gene expression levels of *SOX2* and *NANOG* were higher in the 44ºC HT group than those in the 41ºC HT group. Colony formation assay was performed to compare the clonogenic capacities of the tested groups (Fig. 3c and Fig. S2a). Both HT groups formed more colonies than those in the control (37ºC), and the 44ºC HT group developed 14% more colonies than those in the 41ºC HT group. The senescence-associated β-galactosidase (β-gal) was stained for MSCs in each group on Day 4 (Fig. S2b). Semi-quantitative analyses for the stained areas in the individual groups revealed that β-gal activities were significantly lower in the HT groups, especially HT at 44ºC, than in the control. For example, the β-gal activities of the 41 or 44ºC HT groups were 0.43- and 0.26-fold lower than that of the control group (Fig. 3d). These results suggest that HT enhances the proliferation and suppresses the senescence of MSCs during in vitro culture.

Surface antigen staining is widely performed to quantitatively analyze the stemness and quality of MSCs. For example, MSCs displaying ideal stem cell characteristics are suggested to have ≥ 95% of CD73, CD90, and CD105-positive populations and ≤ 2% of CD14, CD34, and CD45-negative populations [22]. The surface antigen expression of MSCs generally decreases with increasing passage numbers [23]. MSCs with lower passage numbers express more positive markers (such as CD73, CD90, and CD105) and fewer negative markers (such as CD14, CD34, and CD45) than those with higher passage numbers [23]. In our studies, we found that the MSCs in all the groups stained mostly with the positive markers (≥ 99%) and barely with the negative markers (< 1%) (Fig. 3e). The mean fluorescence intensity (MFI) of each surface marker increased with an increase in HT temperature (Fig. 3f). Overall, our experimental results indicate that HT can improve the maintenance and quality of MSC characteristics during in vitro culture, including enhanced proliferation capacity, reduced apoptosis, and delayed aging.

To the best of our knowledge, few studies have clearly demonstrated a correlation between HT and MSC stemness. Using quantitative reverse transcription polymerase chain reaction (RT-PCR) and flow cytometry, our study revealed that HT could promote the expression of HSPs and maintain the stemness of MSCs. We speculated that HSPs may directly and/or indirectly regulate MSC stemness. HSPs are known to play critical roles in regulating stem cell behaviors such as self-renewal, differentiation, and aging [11, 24]. For example, *HSP27*, *HSP70*, and *HSP90* possess cytoprotective functions, such as anti-apoptosis [25], and activate or stabilize STAT3, which controls the expression of key stemness transcription factors (such as *OCT4*, *SOX2*, and *NANOG*) [26–29].

### Heat effects on differentiation potentials of AD-MSCs

**Fig. 4:**
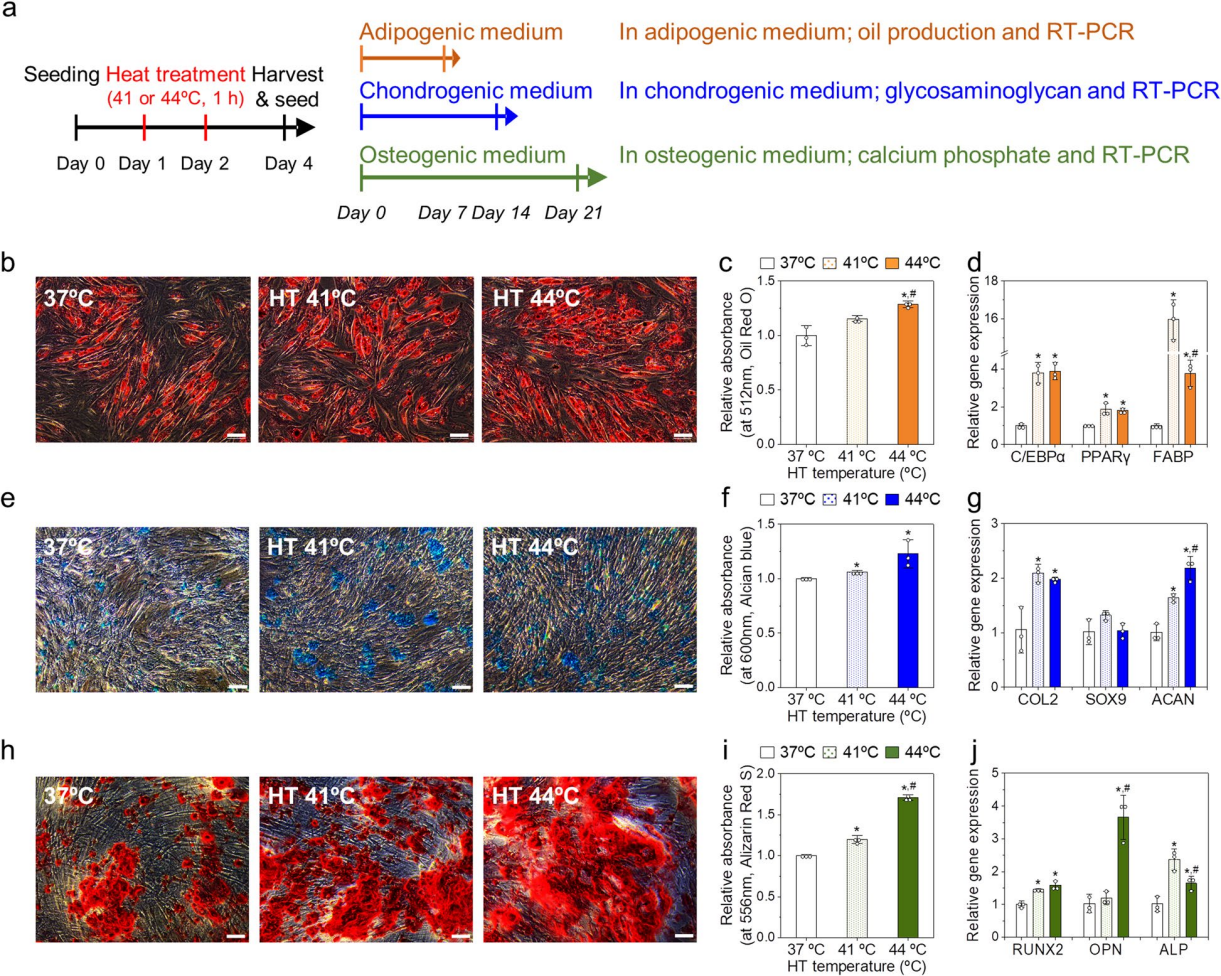
Effects of mild HT on the differentiation capacity of human AD-MSCs. **a** Schematic representation for the experimental design. **b** Oil red-staining images, **c** relative absorbance at 512 nm for Oil Red, and **d** relative expression levels of adipogenic genes (*C/EBPα*, *PPARγ*, and *FABP*) of the MSCs thermally stimulated and cultured in adipogeneic medium for 7 days. **e** Alcian blue-staining images, **f** relative absorbance at 600 nm for Alcian blue, and **g** relative expression levels of chondrogenic genes (*COL2*, *SOX9*, and *ACAN*) of the MSCs thermally stimulated and cultured in chondrogenic medium for 14 days. **h** Alizarin red-staining images, **i** relative absorbance at 556 nm for Alizarin red, and **j** relative expression levels of osteogenic genes (*RUNX2*, *OPN*, and *ALP*) of the MSCs thermally stimulated and cultured in osteogenic medium for 21 days. *p < 0.05 compared to the control group, and ^#^*p* < 0.05 compared to the HT 41ºC group

An important characteristic of MSCs is their ability to differentiate into multiple cell lineages. MSCs typically differentiate into adipocytes, osteoblasts, and chondrocytes depending on the in vitro culture conditions [22]. To investigate the effects of HT on the differentiation potential of MSCs, they were thermally stimulated under the determined HT conditions and subsequently cultured in individual differentiation media (adipogenic, chondrogenic, and osteogenic) for additional 7, 14, and 21 days, respectively (Fig. 4a). HT treatment enhanced the adipogenic differentiation of MSCs, as determined indicated by Oil Red O staining (Fig. 4b, c). Significantly higher oil production was observed in the 41º and 44ºC HT groups with a 1.16- and 1.3-fold increase, respectively, compared to the control group (Fig. 4c). In addition, the expression of adipogenesis-associated genes (*C/EBPα*, *PPARγ*, and *FABP*) was also substantially higher in the HT-treated MSCs than that in the control (Fig. 4d). Similarly, HT treatment also induced the chondrogenic and osteogenic differentiation of MSCs (Fig. 4e-j). Increased calcium deposition and glycosaminoglycan (GAG) production, which are the products of differentiated osteoblasts and chondrocytes, respectively, were observed in the HT-treated groups compared to those in the control groups (Fig. 4f, i). Moreover, GAG production and calcium deposition in the 44ºC HT groups were 1.2- and 1.4-fold higher than those in the 41ºC groups, respectively. The expression levels of chondrogenic and osteogenic genes were higher in the HT groups than those in the control group, although the effects of HT temperature (41 or 44ºC) on gene expression were not clearly significant (Fig. 4g, j). Overall, we observed that the differentiation of MSCs into the three lineages was significantly potentiated by HT, which may be attributed to the upregulation of HSPs. Several studies have reported hyperthermia-induced enhancement of MSC differentiation. For example, human bone marrow-derived MSCs showed enhanced osteogenesis after mild HT [30]. Our results also indicated that HT significantly improves the differentiation potential of MSCs. In particular, specific HT conditions (2X HT at 41 or 44ºC for 1 h) positively affect stemness maintenance and differentiation potential in a single passage; using these conditions, we further examined the feasibility of HT for long-term MSC culture.

### Lasting effects on HT on AD-MSCs

**Fig. 5:**
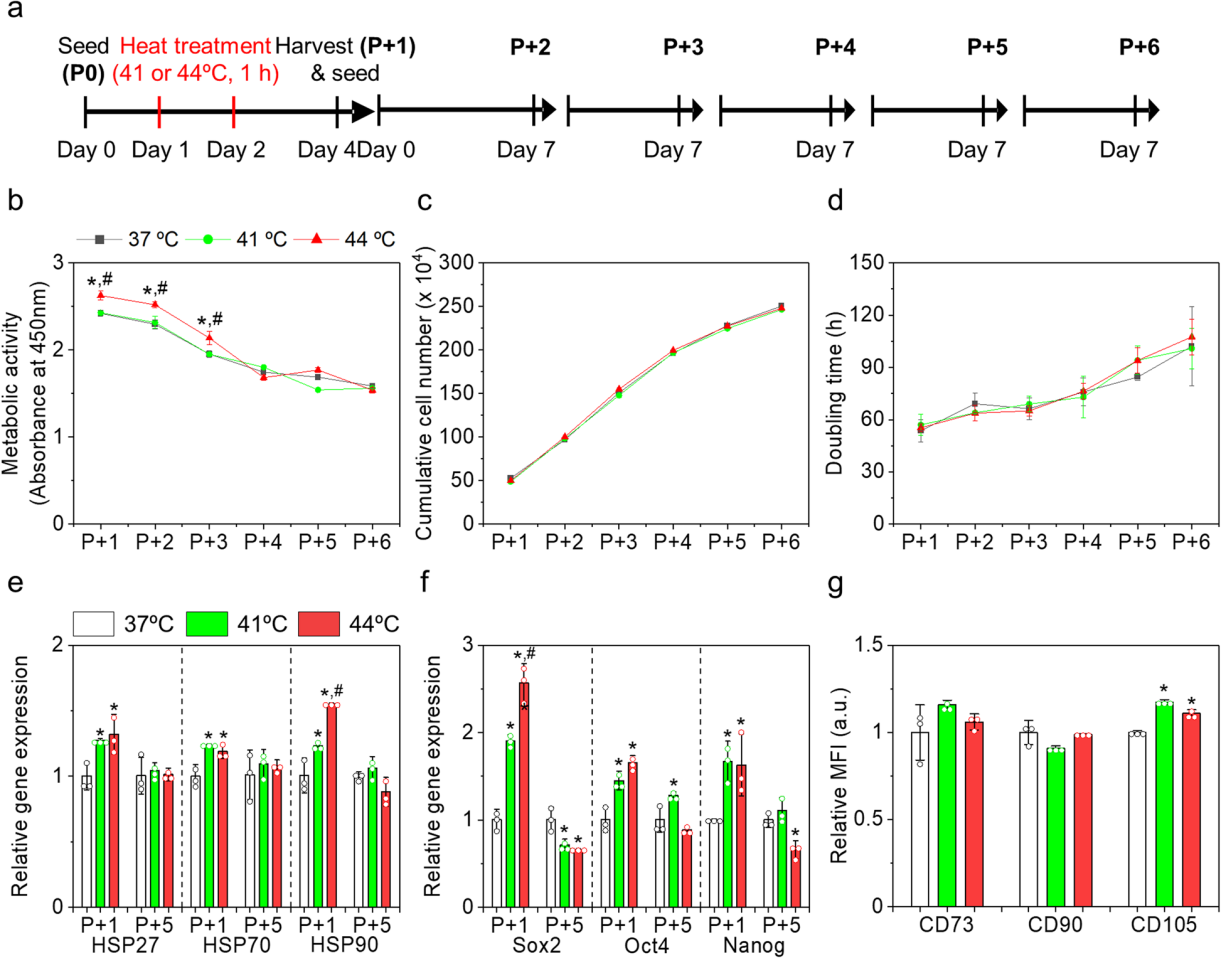
Lasting effects of mild HT on human AD-MSCs during subculturing without additional HT. **a** Schematic representation of the experimental design. **b** metabolic activity, **c** cumulative cell number, and **d** doubling time of the MSCs in each group during the subculture. The relative gene expressions of **e** HSPs (*HSP27*, *HSP70*, and *HSP90*) and **f** stemness markers (*SOX2*, *OCT4*, and *NANOG*) in each group. **g** Relative MFI of the positive marker-stained MSCs in each group at P + 6. MFI was obtained using flow cytometry. *p < 0.05 compared to the control group, and ^#^*p* < 0.05 compared to the HT 41ºC group

The positive effect of HT on MSC quality (stemness and differentiation potential) was demonstrated in a single passage. To further investigate the duration of this effect, we sub-cultured MSCs for up to five additional passages without providing additional HT during incubation (Fig. 5a). The passage number was denoted as P + *n* after each subculture, where *n* indicates the number of subcultures. After five passages, no morphological differences were observed between the control and HT groups, and the MSCs in all the groups were spindle-shaped (Fig. S3a). Metabolic activity in all the groups gradually decreased during incubation. MSCs in the HT 41ºC groups showed metabolic activities similar to that in the control group (Fig. 5b). Interestingly, at P + 1, P + 2, and P + 3, metabolic activities were approximately 10% higher for the 44ºC HT-treated MSCs than those of the control and the 41ºC HT-treated MSCs. However, this effect diminished and became similar to that of the other groups after the third passage. Thus, the effect of HT, particularly at 44ºC, might last for additional two generations and eventually disappear. The cumulative cell number and doubling time were not significantly different among the groups (Fig. 5c, d). We compared the gene expression of HSPs and stem cell transcription factors between MSCs at P + 1 (HT-treated MSCs) and P + 6 in the individual groups (Fig. 5e, f). Overall, the expression levels were higher in HT-treated MSCs than those in control MSCs at P + 1, but were not significantly different among the groups at P + 6. We observed that the HT 41 °C group at low passage numbers showed metabolic activity similar to the control group, although the expression level of HSPs in the HT 41 °C group was higher than that in the control group. We speculate that a single HT at 41ºC might result in insufficient expression of HSPs to stimulate cellular activity within a few passage numbers [31].

Surface antigen staining revealed that almost 100% of the MSC population in all the groups at P + 6 still presented with positive markers and < 2% of the population presented with negative markers (Fig. S3b). Except for CD105, the relative MFIs of the positive marker-stained MSCs were not significantly different in the tested groups (Fig. 5g). Results indicated that HT history did not have a detrimental impact on the stemness of MSCs and HT effects could last for a maximum of two additional passages.

### Long-term subculture of MSCs with HT

**Fig. 6:**
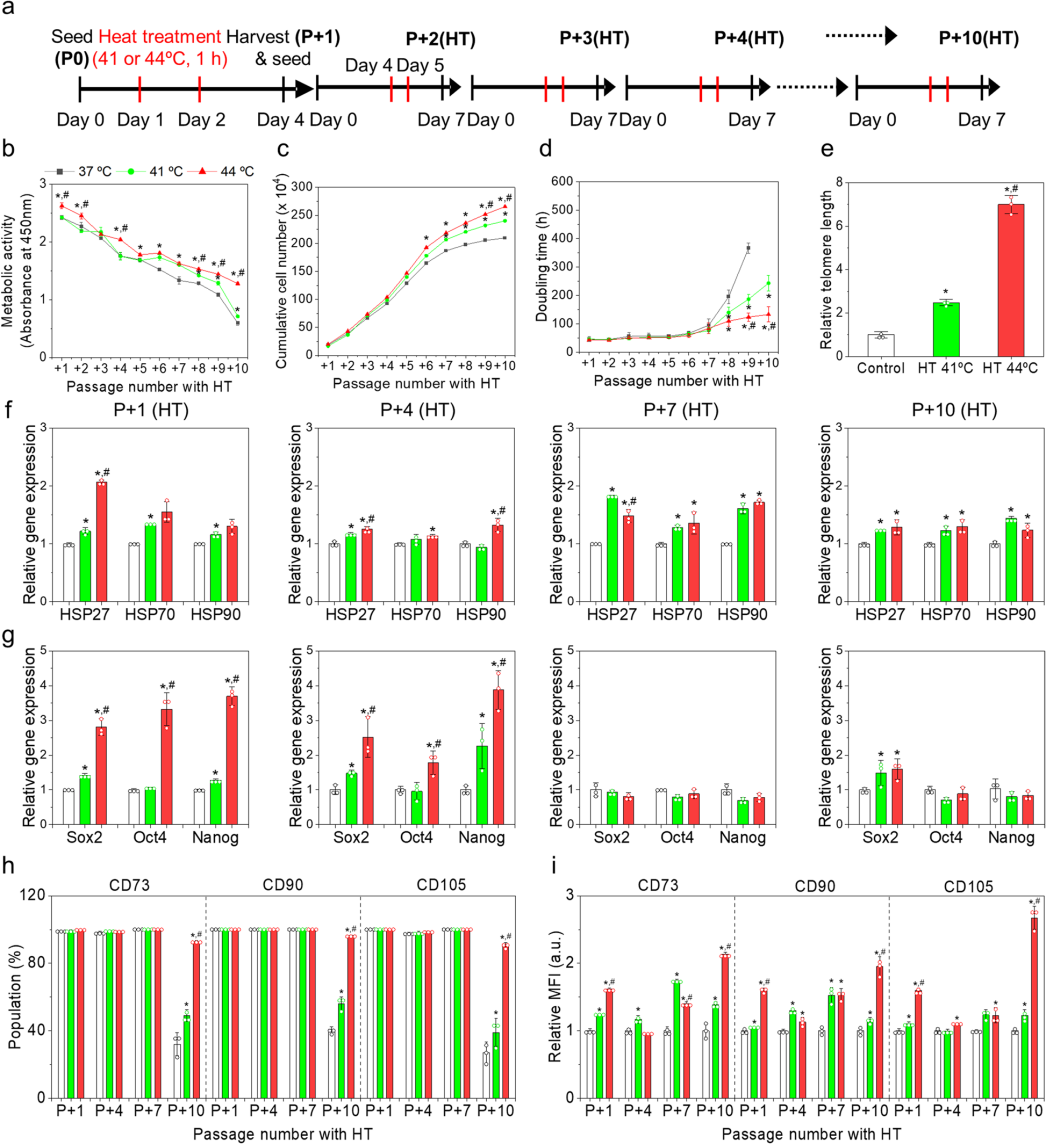
Effects of successive mild HT on human AD-MSCs during subculturing with HT. **a** Schematic representation of the experimental study design. **b** Metabolic activity, **c** cumulative cell number, and **d** doubling time of the MSCs in each group during the subculture. **e** Telomere lengths of the MSCs at P + 10(HT) in each group. Relative gene expressions of **f** HSPs (*HSP27*, *HSP70*, and *HSP90*) and **g** stemness markers (*SOX2*, *OCT4*, and *NANOG*) at P + 1(HT), P + 4(HT), P + 7(HT), and P + 10(HT) in each group. Gene expression levels were normalized with those of the control at each passage. **h** Populations and **i** MFI of the positive marker-stained MSCs in each group. *p < 0.05 compared to the control group, and ^#^*p* < 0.05 compared to the HT 41ºC group

We periodically subjected the MSCs to HT at each passage, to examine the possibility of expanding and potentiating MSCs for long-term culture with reduced aging, since the effect of HT on MSCs in a single passage diminished with subsequent passages. MSCs were subcultured with HT (41 or 44ºC) up to additional tenth passage (P + 10(HT)) (Fig. 6a). No morphological differences were observed between the control and HT groups after four passages (P + 4(HT)). However, MSCs showed a large, flattened, and irregular shape in all the groups from P + 7(HT), which was distinct from the control group (Fig. S4a). In addition, the control group exhibited a dramatically reduced number of cells. The metabolic activities of MSCs decreased over time in all the groups. For example, the MSCs in the control group had 70 and 25% of their initial metabolic activities at P + 5(HT) and P + 10(HT), respectively. Metabolic activities in the 44ºC HT group moderately decreased and remained higher than those in the control group for most passages (Fig. 6b). On the other hand, MSCs in the 41ºC HT group showed higher metabolic activities than those in the control at high passage numbers (> P + 5(HT)), but their metabolic activities were very low at P + 10(HT). At P + 10(HT), the metabolic activities of the control, 41ºC HT, and 44ºC HT groups were 25 ± 5%, 30 ± 3%, and 41 ± 6% of their initial activities at P + 1(HT), respectively. Cumulative cell number analyses revealed more MSCs in the HT groups than those in the control groups throughout the subculture, wherein the 44ºC HT groups had higher cumulative cell numbers than those in the 41ºC HT group (Fig. 6c). Increases in cumulative cell numbers were linear in all groups up to P + 6(HT), and then gradually slowed. In the HT 44ºC groups, the decreases in cumulative cell numbers were relatively moderate. The cumulative cell numbers at P + 10(HT) were 2.1, 2.4, and 2.7 × 10^6^ cells in the control, 41ºC HT, and 44ºC HT groups, respectively. Doubling times slowly and gradually increased up to P + 6(HT) in all groups and were not significantly different among groups. MSCs in the HT 41ºC and control group showed dramatic increases in doubling times after P + 6(HT) (Fig. 6d). In contrast, the HT 44ºC group exhibited a slow increase in doubling time to P + 10(HT), with no abrupt increase and a shorter doubling time compared to the other groups. Doubling times of the control, 41ºC HT, and 44ºC HT groups at P + 9(HT) were 367 ± 18, 187 ± 17, and 124 ± 14 h, respectively. Overall, HT using ideal conditions (44ºC, 1 h per day, and 2X) could improve cell proliferation for long-term culture, allowing for extended passage numbers, although successive subculture of MSCs, even with HT, eventually led to aging with impaired proliferation ability. We measured the telomere lengths of the MSCs in each group at P + 10(HT) (Fig. 6e). Surprisingly, the telomeres of the MSCs in the HT 41ºC and the HT 44ºC groups were 2.5-fold and 7.0-fold longer, respectively, than that of the control, implying that HT could retard aging of the MSCs cultured in vitro. Long telomere in the MSCs in the HT groups implies that HT can support the maintenance of stemness and the proliferation of MSCs.

Gene expression of HSPs was higher in the HT groups than those in the control throughout the passages up to P + 10(HT), implying that MSCs responded to HT even in the aged state (Fig. 6f). Expression of some HSP genes (*HSP27* and *HSP90*) at P + 1(HT) and P + 4(HT) was higher in the HT 44ºC group than those in the HT 41ºC group. However, after P + 4(HT), HSP expression levels were mostly similar in the HT groups. The expression of stemness genes was significantly higher in the HT 44ºC group than those in the HT 44ºC and control groups up to P + 4(HT), and such differences were not significant among all groups from P + 7(HT) (Fig. 6g). Flow cytometry with surface antigen staining demonstrated that MSCs in all the groups up to P + 7(HT) maintained stem cell characteristics, with > 99% positive and < 1% negative markers. However, at P + 10(HT), a significantly decreased population of positive marker stained MSCs were observed from the control (26–42%) and HT 41ºC (40–56%) groups, whereas the MSCs in the HT 44ºC groups still presented high populations with positive marker-staining (> 90%) (Fig. 6h and Fig. S4b). The MFI values of positive marker-stained cells were consistently higher in the HT group than those in the control group throughout the passages up to P + 10(TH) (Fig. 6i). The HT 44ºC group consistently showed high intensities of positive surface markers. Altogether, although HT could not prevent in vitro aging of cultured MSCs, HT particularly at 44ºC could reduce MSC aging and maintain its stem cell characteristics during relative long-term in vitro culture. For the long-term culture, we started culture of MSCs at passage 5 as relatively aged cells. However, the younger MSCs at relatively low passage number would respond to HT more or less dramatically, which would be necessary as a future work.

### Heat effects on differentiation potential of AD-MSCs in long-term subculture with HT

**Fig. 7:**
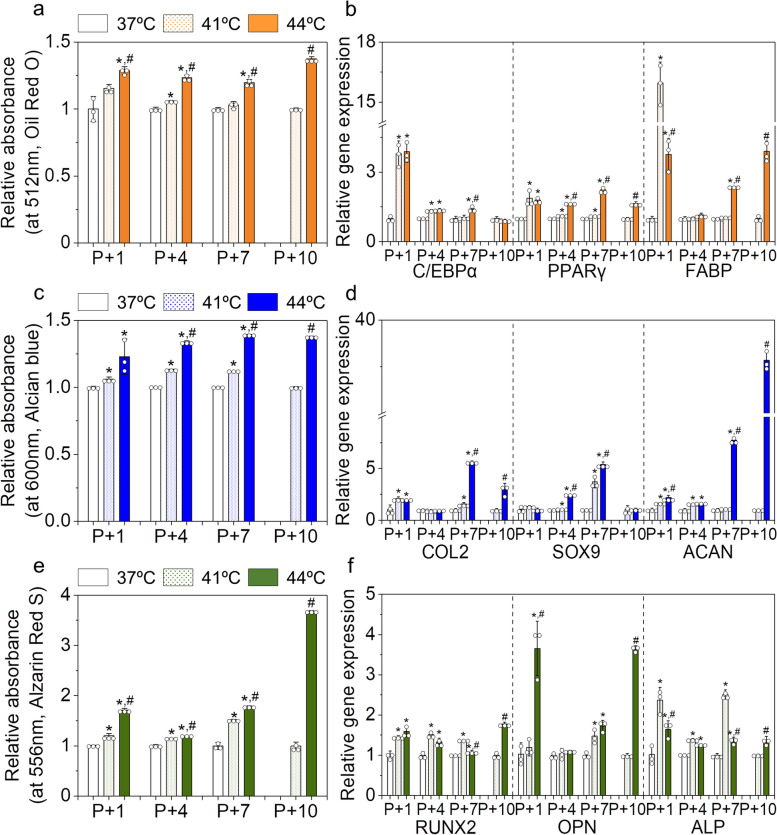
Effects of mild HT on the differentiation capacity of human AD-MSCs during subculturing with HT. **a** Relative absorbance of oil red and **b** relative expression levels of adipogenic genes (*C/EBPα*, *PPARγ*, and *FABP*) of the MSCs in each group during the subculture at P + 1(HT), P + 4(HT), P + 7(HT), and P + 10(HT). **c** Relative absorbance of Alcian blue and **d** relative expression levels of chondrogenic genes (*COL2*, *SOX9*, and *ACAN*) of the MSCs in each group during the subculture. **e** Relative absorbance of Alizarin red and **f** relative expression levels of osteogenic genes (*RUNX2*, *OPN*, and *ALP*) of the MSCs in each group during the subculture. The absorbance and gene expression levels were normalized with those of the control at each passage. *p < 0.05 compared to the control group, and ^#^*p* < 0.05 compared to the HT 41ºC group

We further examined the differentiation potential of MSCs after subculturing them with HT. To this end, we cultured MSCs obtained from each group at specific passages in each differentiation medium (adipogenic, chondrogenic, and osteogenic medium). Note that differentiation tests for MSCs obtained at P + 10(HT) in the control group could not be performed because of very low cell numbers and low growth rates. We found that HT significantly improved the differentiation potential of MSCs into all three lineages at all passages during long-term subculturing with HT. More differentiation products (such as oil, glycosaminoglycan, and calcium phosphate) were obtained in the HT group than those in the control group throughout the test passages (Fig. 7a, c, and e). The HT 44ºC group showed the most significant improvement. For example, MSCs from the HT 44ºC group at P + 10(HT) produced 1.4-fold more oil red compared to those from the HT 41ºC group (Fig. 7a). In addition, calcium deposition was1.5- and 1.8-fold higher in the HT 41 and 44ºC groups than that in the control group at P + 7(HT) (Fig. 7e). The expression of specific differentiation genes in MSCs cultured in individual differentiation media was higher in the HT group than that in the control group (Fig. 7b, d, f, and Fig. S5). These results suggest that successive HT could enhance the differentiation potential of MSCs, even at a high passage number. HT may retard the aging process and/or promote MSC differentiation.

Our results demonstrated that long-term subculture with HT, particularly at 44ºC, could improve the maintenance of stemness, growth, and differentiation potential of MSCs. This HT-induced improvement in MSC quality can be attributed to the upregulation of HSP expression during short-and long-term culture. HSPs are expressed in response to external stresses and play important roles in maintaining the viability, function, and differentiation of multiple cell types. For example, Andreeva et al. reported that exogenous *HSP70* and mid-HT promoted the growth of aged MSCs [31]. Our experimental results suggest that HT is a promising strategy for producing high-quality MSCs, even with increased passages.

### RNA sequencing

**Fig. 8:**
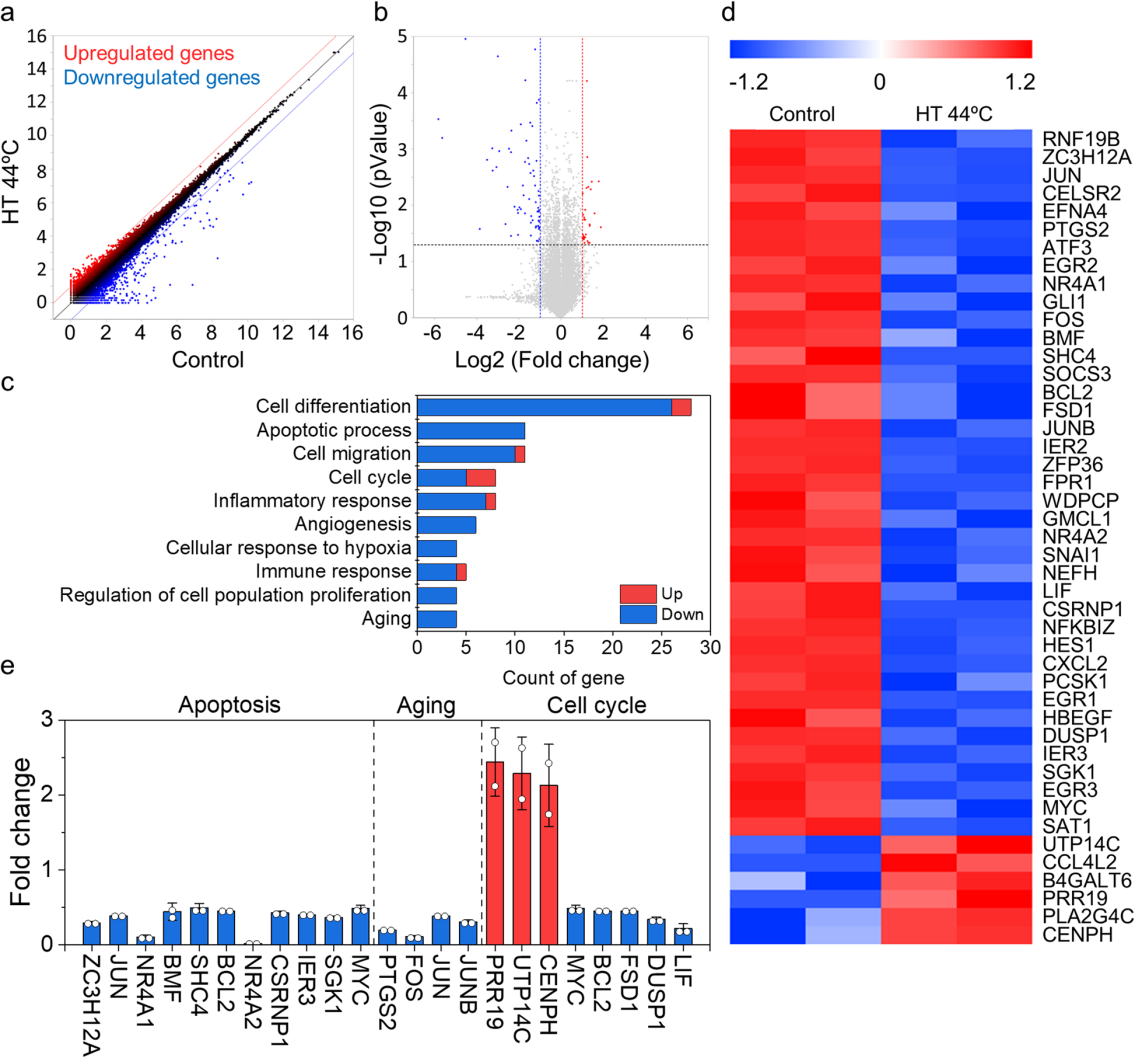
Total RNA sequencing analysis. **a** Scatter plot. The red and blue dots indicate up-regulated and down-regulated genes, respectively, of the 44ºC heat treated-MSCs compared to the control. **b** Volcano plot. **c** Ten major gene ontologies closely related with the genes, which showed significantly altered expressions induced by HT. **d** Heat map of the two repeated analysis for each gene which are involved in the listed gene ontologies. **e** Specific gene expression profiles of each gene ontology; apoptosis, aging, and cell cycle

Quantitative RNA sequencing (Quant-Seq) was performed to investigate the mechanisms by which HT influences MSCs. We compared RNA expressions in the control and the 44ºC HT group and found substantial changes in the expression of total 25,737 genes (Fig. 8a). In particular, the expression of 101 genes in the HT group was two-fold or higher (*p* < 0.05) than that in the control group (Fig. 8b); 30 genes were upregulated and 71 genes were downregulated in the HT group. Gene ontology analysis identified 10 major ontologies, including 54 genes associated with these identified genes (Fig. 8c). The heat map showed that the expression of these 54 genes was substantially upregulated or downregulated by HT (Fig. 8d). We found that HT influenced apoptosis, aging, and the cell cycle in cultured MSCs (Fig. 8e). Furthermore, we identified 11, 4, and 8 significantly altered genes in the apoptosis, aging, and cell cycle categories, respectively. Eleven apoptosis-related genes were downregulated in the HT group compared with those in the control group. Some genes (such as *MYC*, *IER3*, *CSRNP1*, *BMF*, *JUN*, and *ZC3H12A*) involved in inducing apoptosis were downregulated, suggesting that HT may inhibit apoptosis and promote cell proliferation [32–37]. Additionally, the following four genes related to aging were downregulated in the HT group: *FOS*, *JUN*, *JUNB,* and *PTGS2*. These genes were reported to induce cell aging and their expression levels are typically high in aged cells [38–40]. Regarding cell cycle, the up-regulated (*CENPH*, *UTP14C*, and *PRR19*) and down-regulated (*LIF*, *DUSP1*, *FSD1*, *BCL2*, *MYC*) genes were identified. *CENPH*, *UTP14C*, and *PRR19* are involved in cell cycle progression. *DUSP1* and *BCL2* are known to suppress cell-cycle progression [41–45]. These findings suggest that HT influences various MSC functions, including apoptosis, aging, and cell cycles, which in turn enhance MSC qualities, such as stemness maintenance, proliferation, and differentiation potentials, and delay the aging process.

## Discussion

We aimed to systematically investigate the effects of HT on MSCs and provide a comprehensive understanding of the optimal HT conditions for MSC culture. We evaluated the impact of HT on various aspects of MSCs, including stemness, differentiation potential, long-term subculture, and underlying mechanisms. Firstly, we found that mild HT at temperatures of 41 and 44ºC for 1 h consistently upregulated HSPs and stemness-associated transcription factors in MSCs without compromising cell viability, suggesting that HT can induce a beneficial heat shock response in MSCs, enhancing their stemness and self-renewal capacity. Additionally, we investigated the effects of HT on the differentiation potential of MSCs and found that HT under the determined conditions significantly enhanced the adipogenic, chondrogenic, and osteogenic differentiation of MSCs. HT-treated MSCs exhibited increased production of lineage-specific differentiation products and higher expression of genes associated with adipogenesis, chondrogenesis, and osteogenesis. These findings highlight the potential of HT to promote and potentiate the differentiation of MSCs into specific lineages, which is crucial for their therapeutic applications in regenerative medicine. Furthermore, we employed successive HT for MSCs during long-term subculture. HT-treated MSCs maintained their metabolic activity, cumulative cell numbers, and stemness characteristics for a longer duration compared to the control group. The expression of HSPs remained higher in HT-treated MSCs, indicating the sustained influence of HT on MSCs. This suggests that HT has the potential to enhance the quality and longevity of MSCs during long-term culture, which is essential for their clinical application. Based on quantitative RNA sequencing, we identified significant changes in the expression of thousands of genes, with specific pathways related to apoptosis, aging, and the cell cycle being influenced by HT. The HT downregulated apoptosis-related and aging-related genes, suggesting that HT may inhibit apoptosis, delay the aging process, and promote cell proliferation. Furthermore, HT affected the expression of genes involved in cell cycle regulation, indicating its potential to modulate cell cycle progression and proliferation.

This comprehensive study provides valuable insights into the optimal HT conditions for in vitro MSC culture and sheds light on the multifaceted effects of HT on MSCs. The findings demonstrate that HT can enhance the long-term maintenance of MSC stemness. These results have important implications for the development of strategies to improve the quality and therapeutic potential of MSCs in regenerative medicine. Further research on the specific molecular mechanisms underlying the effects of HT on MSCs is necessary to fully elucidate its therapeutic potential and optimize its application in clinical settings.

## Conclusion

In this study, we aimed to develop an in vitro culture system for producing high-quality and large-quantity MSCs. Therefore, we examined the effects of specific HT conditions on MSC characteristics in both short- and long-term subcultures. Particularly, 1 h per day HT at 41 or 44ºC, on both day 1 and day 2 was found to up-regulate the expression of HSPs and stemness markers and promote the growth and differentiation potentials of MSCs without hampering their viability. Furthermore, successive HT during subculture up to additional 10 passages identified that HT at 44ºC effectively reduced the loss of MSC characteristics, such as growth, stemness, and differentiation potential. RNA sequencing analyses revealed that HT significantly altered the expression of various genes, particularly those associated with apoptosis, aging, and cell cycle. The HT-induced upregulation of these genes may play a potential role in promoting MSC qualities (such as stemness maintenance). In conclusion, our study successfully demonstrated that mild HT of MSCs benefits their utilization in various biomedical fields by potentiating MSCs and retarding aging.

### Supplementary Information

**Additional file 1:** **Supplementary Fig. 1.** The optical images for the heat treatment (HT) of MSCs. a The optical images of MSCs for HT at temperature 41 and 44ºC for 0.5, 1, and 2 h. b The optical images of MSCs for HT at temperature 41 and 44ºC for 1 h once (1X) and twice (2X). Scale bars are 200 μm. **Supplementary Fig. 2.** Optical images for colony formation assay and ß-gal staining. a The images of stained colonies formed by MSCs in six-well plates. b The images of ß-gal-stained MSCs in 12-well plates. Scale bars are 200 μm. **Supplementary Fig. 3.** The lasting effect. a The optical images of MSCs at each passage. b The population of each surface marker-stained MSCs at P+6 using flow cytometry. Scale bars are 200 μm. **Supplementary Fig. 4.** Periodic HT. a The optical images of heat treated-MSCs at each passage. b The population of surface marker-stained MSCs at each passage using flow cytometry. Scale bars are 200 μm. **Supplementary Fig. 5.** Periodic HT. The stained images of trilineage differentiation at a P+4, b P+7, and c P+10. Scale bars are 200 μm. **Table S1.** Primer sequences for qRT-PCR.

## Data Availability

The data that support the findings of this study are available from the corresponding author upon reasonable request.
