# Supplementary material for: Mild heat treatment in vitro potentiates human adipose stem cells: delayed aging and improved quality for long term culture
Source: Biomater Res. 2023 Nov 27;27:122. doi: 10.1186/s40824-023-00448-w (PMC10680349; doi:10.1186/s40824-023-00448-w)
Supplement: Supplementary file 1 — Additional file 1: Supplementary Fig. 1. The optical images for the heat treatment (HT) of MSCs. a The optical images of MSCs for HT at temperature 41 and 44ºC for 0.5, 1, and 2 h. b The optical images of MSCs for HT at temperature 41 and 44ºC for 1 h once (1X) and twice (2X). Scale bars are 200 μm. Supplementary Fig. 2. Optical images for colony formation assay and ß-gal staining. a The images of stained colonies formed by MSCs in six-well plates. b The images of ß-gal-stained MSCs in 12-well plates. Scale bars are 200 μm. Supplementary Fig. 3. The lasting effect. a The optical images of MSCs at each passage. b The population of each surface marker-stained MSCs at P+6 using flow cytometry. Scale bars are 200 μm. Supplementary Fig. 4. Periodic HT. a The optical images of heat treated-MSCs at each passage. b The population of surface marker-stained MSCs at each passage using flow cytometry. Scale bars are 200 μm. Supplementary Fig. 5. Periodic HT. The stained images of trilineage differentiation at a P+4, b P+7, and c P+10. Scale bars are 200 μm. Table S1. Primer sequences for qRT-PCR. [file 40824_2023_448_MOESM1_ESM.docx]

**Supplementary information**

Mild heat treatment in vitro potentiates human adipose stem cells: delayed aging and improved quality for long term culture

*Chiseon Ryu^1^, Minseo Lee^1^ and Jae Young Lee^1,^**

^1^School of Materials Science and Engineering, Gwangju Institute of Science and Technology, Gwangju, 61005, Republic of Korea

*jaeyounglee@gist.ac.kr

**
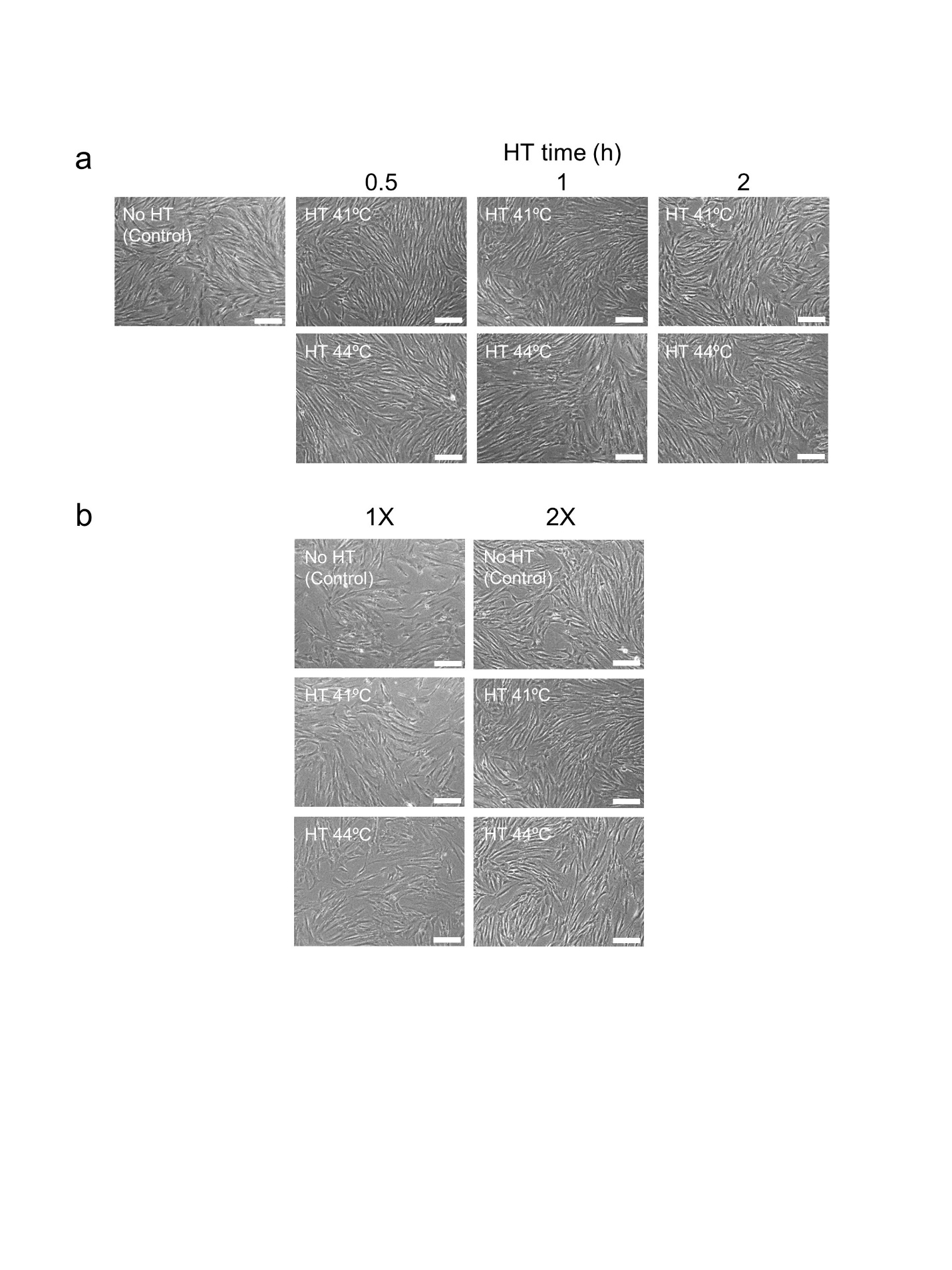
**

**Supplementary Fig. 1: The optical images for the heat treatment (HT) of MSCs. a** The optical images of MSCs for HT at temperature 41 and 44ºC for 0.5, 1, and 2 h. **b** The optical images of MSCs for HT at temperature 41 and 44ºC for 1 h once (1X) and twice (2X). Scale bars are 200 μm.


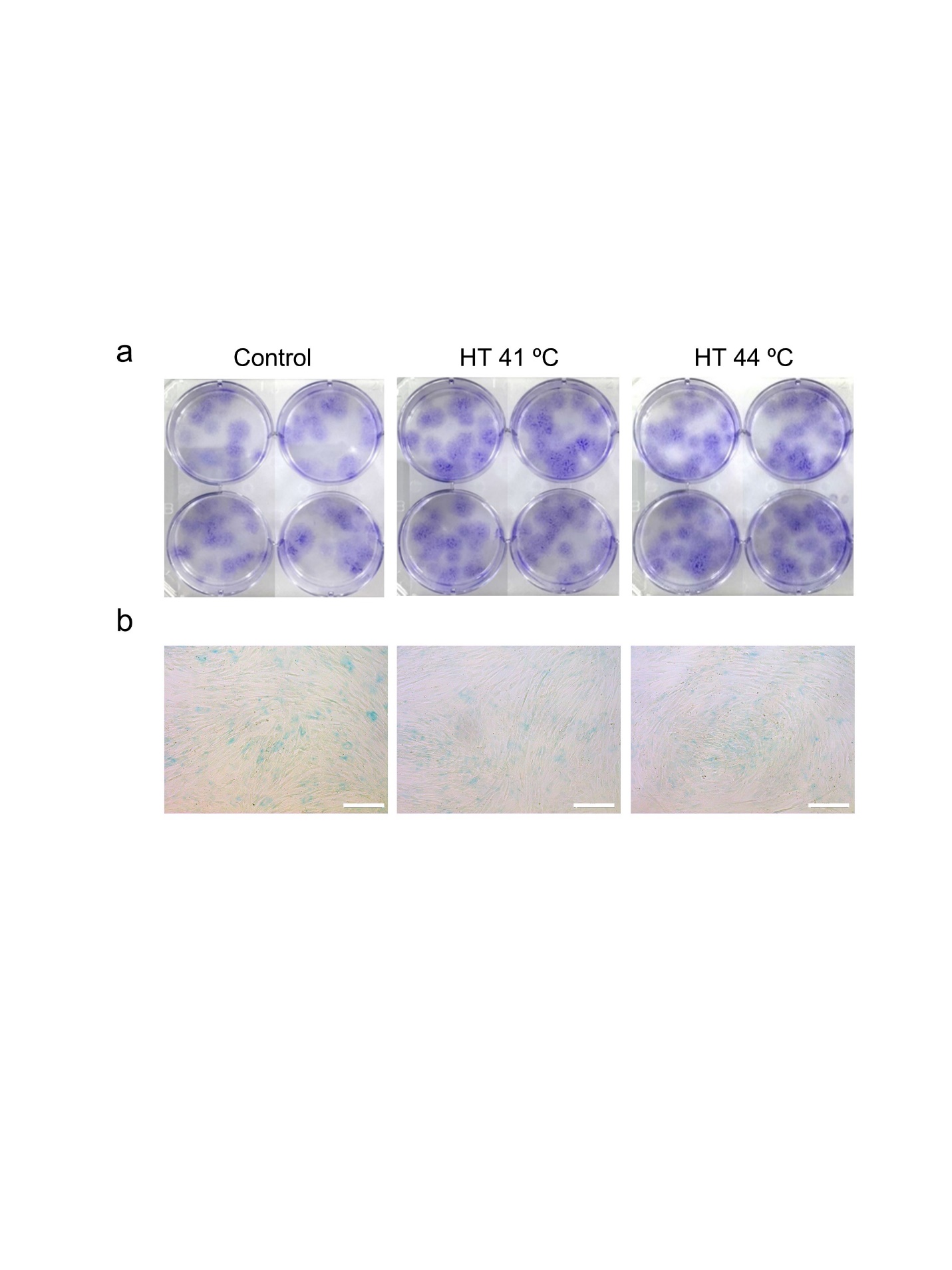


**Supplementary Fig. 2: Optical images for colony formation assay and ß-gal staining. a** The images of stained colonies formed by MSCs in six-well plates. **b** The images of ß-gal-stained MSCs in 12-well plates. Scale bars are 200 μm.


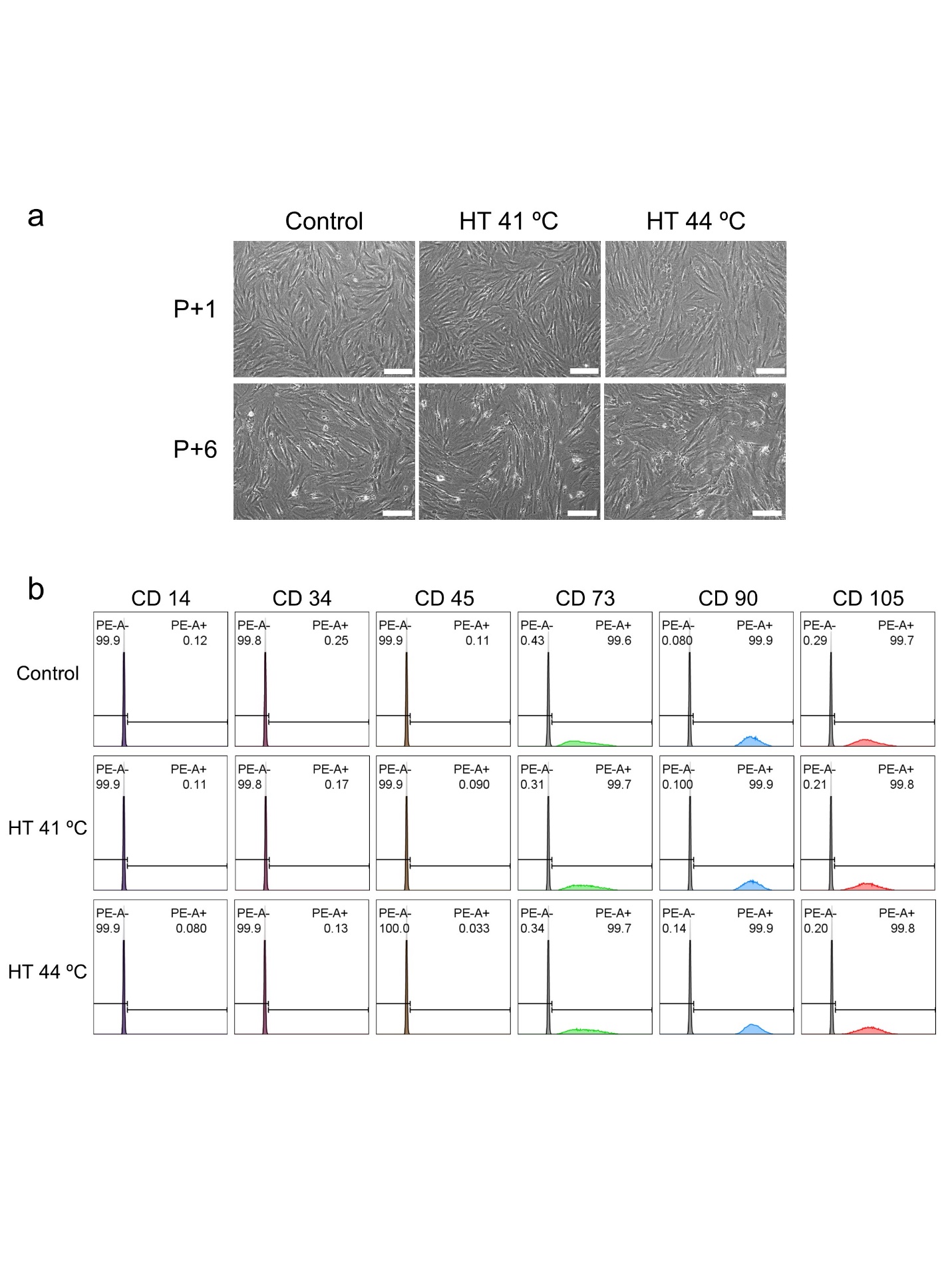


**Supplementary Fig. 3: The lasting effect. a** The optical images of MSCs at each passage. **b** The population of each surface marker-stained MSCs at P+6 using flow cytometry. Scale bars are 200 μm.


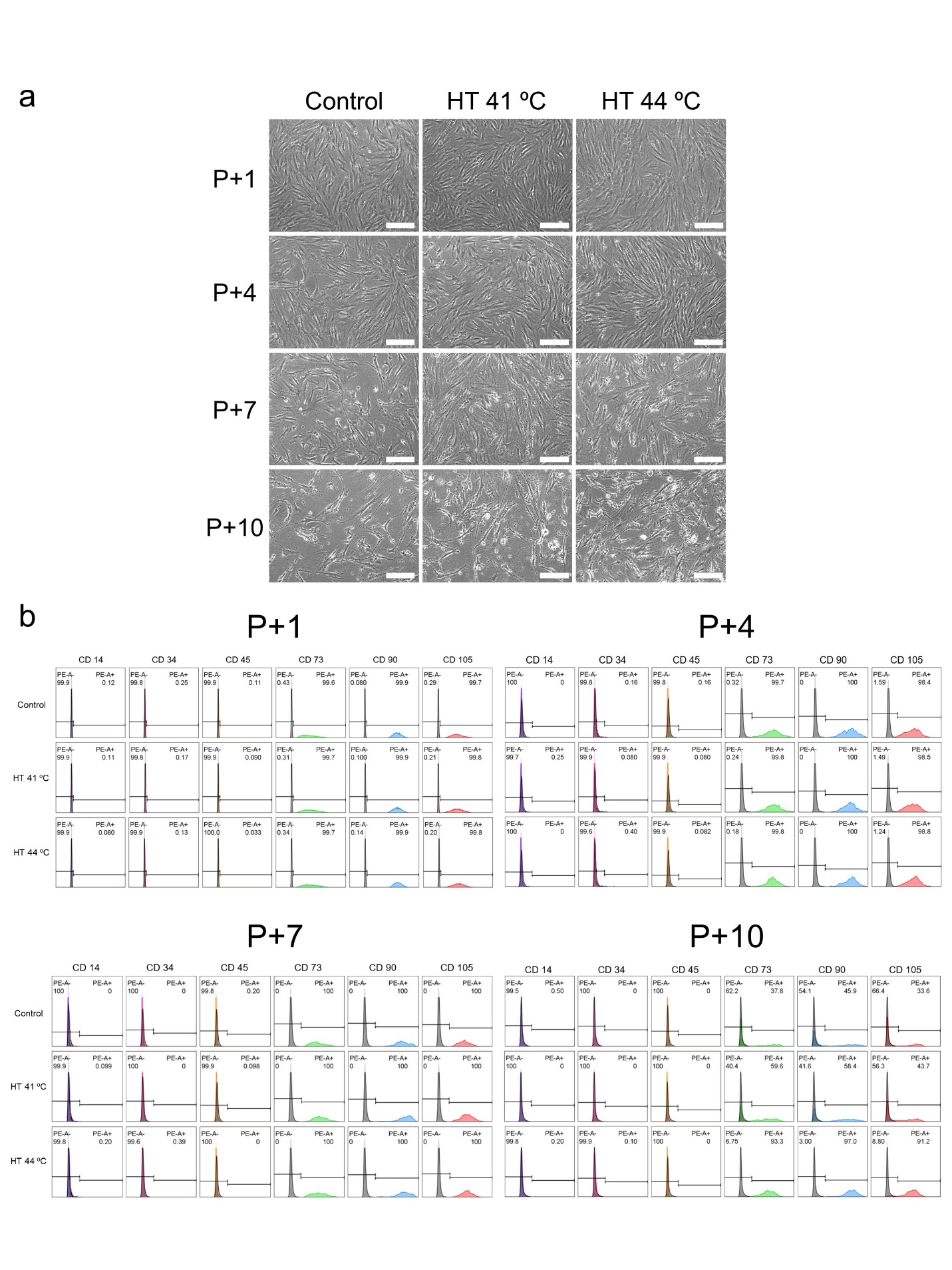


**Supplementary Fig. 4: Periodic HT. a** The optical images of heat treated-MSCs at each passage. **b** The population of surface marker-stained MSCs at each passage using flow cytometry. Scale bars are 200 μm.

**
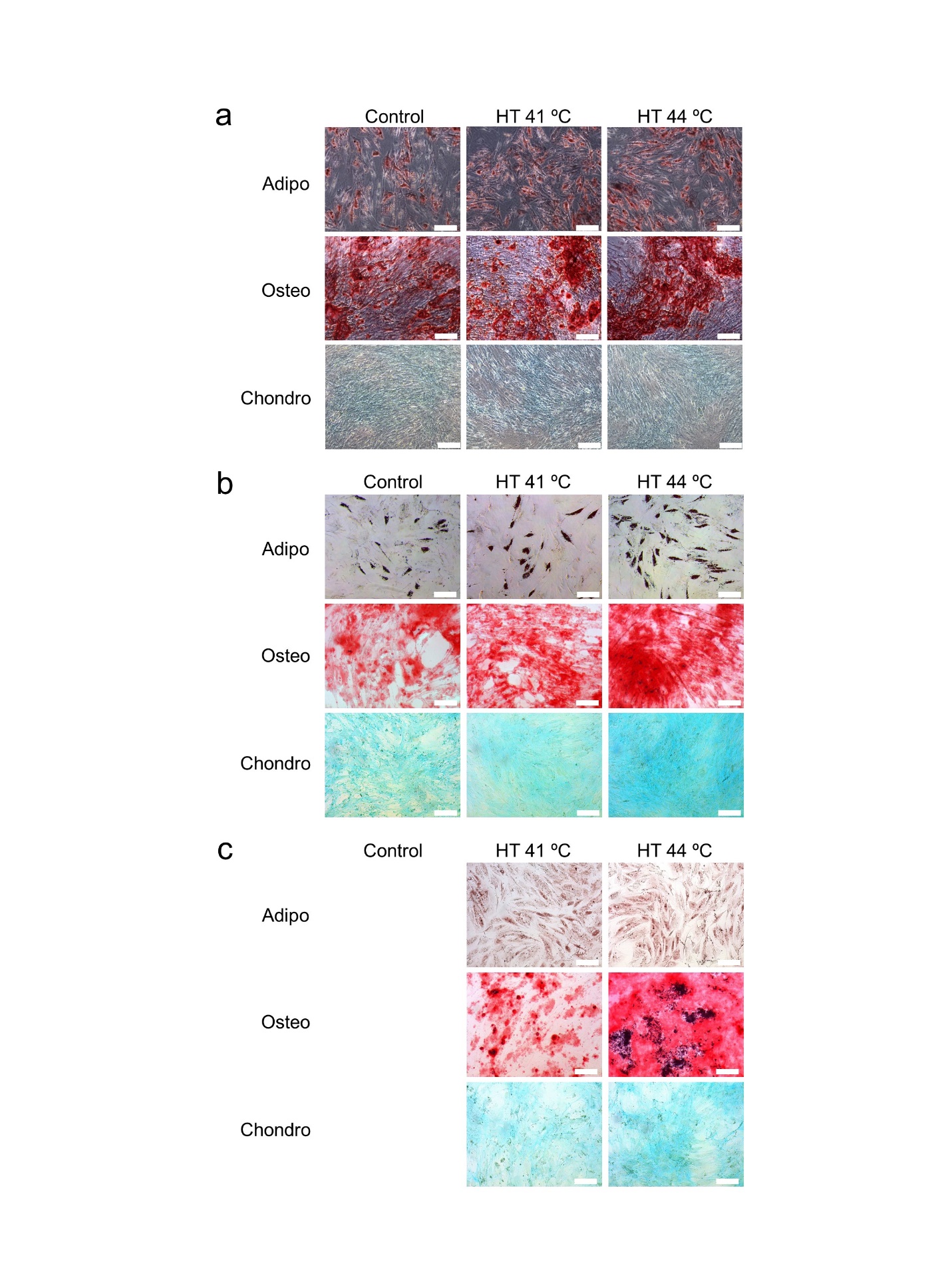
**

**Supplementary Fig. 5: Periodic HT.** The stained images of trilineage differentiation at **a** P+4, **b** P+7, and **c** P+10. Scale bars are 200 μm.

| ***Gene*** | ***Forward primer (5′-3′)*** | ***Reverse primer (5′-3′)*** |
| --- | --- | --- |
| *GAPDH* | ATTTGGTCGTATTGGGCG | TGGAAGATGGTGATGGGATT |
| *HSP27* | GTCCAACGAGATCACCATCC | CGGCAGTCTCATCGGATTT |
| *HSP70* | TTGCAGTGTGCCATCTTATC | GAGAAAGGAGCAGCATGATT |
| *HSP90* | CACCACTCTACTCTGTCTCT | GTTTCCTCAGGCATCAGTAG |
| *SOX2* | TGCGAGCGCTGCACAT | GCAGCGTGTACTTATCCTTCTTCA |
| *OCT4* | ACATCAAAGCTCTGCAGAAA | CTGAATACCTTCCCAAATAGAAC |
| *NANOG* | AATACCTCAGCCTCCAGCAGAT | TGCGTCACACCATTGCTATTCTT |
| *C/EBPΑ* | CGGTGGACAAGAACAGCAAC | CGGAATCTCCTAGTCCTGGC |
| *PPARΓ* | TGTCTCATAATGCCATCAGGTTTG | GATAACGAATGGTGATTTGTCTGTT |
| *FABP* | ATGCTTTTGTAGGTACCTGG | CTCTCTCATAAACTCTCGTG |
| *RUNX2* | GGCCCACAAATCTCAGATCGTT | CACTGGCGCTGCAACAAGAC |
| *OPN* | TTGCAGCCTTCTCAGCCAA | GGAGGCAAAAGCAAATCACTG |
| *ALP* | AACACCACCCAGGGGAAC | GGTCACAATGCCCACAGATT |
| *COL2A1* | CCGGGCAGAGGGCAATAGCAGGTT | CAATGATGGGGAGGCGTGAG |
| *SOX9* | AGCGAACGCACATCAAGAC | CTGTAGGCGATCTGTTGGGG |
| *ACAN* | CTACCGCTGCGAGGTGATG | TCGAGGGTGTAGCGTGTAGAGA |
| *TEL1* | GGTTTTTGAGGGTGAGGGTGAGGGTGAGGGTGAGGGT | |
| *TEL2* | TCCCGACTATCCCTATCCCTATCCCTATCCCTATCCCTA | |
| *36B4U* | CAGCAAGTGGGAAGGTGTAATCC | |
| *36B4D* | CCCATTCTATCATCAACGGGTACAA | |

**Table S1: Primer sequences for qRT-PCR.**
